# Diffusivity of the uncinate fasciculus in heroin users relates to their levels of anxiety

**DOI:** 10.1038/tp.2015.48

**Published:** 2015-04-28

**Authors:** N M L Wong, S-H Cheung, C C H Chan, H Zeng, Y-P Liu, K-F So, T M C Lee

**Affiliations:** 1 Laboratory of Neuropsychology, The University of Hong Kong, Hong Kong, Hong Kong; 2 Laboratory of Social Cognitive Affective Neuroscience, The University of Hong Kong, Hong Kong, Hong Kong; 3 Institute of Clinical Neuropsychology, The University of Hong Kong, Hong Kong, Hong Kong; 4 Department of Psychology, The University of Hong Kong, Hong Kong, Hong Kong; 5 Applied Cognitive Neuroscience Laboratory, The Hong Kong Polytechnic University, Hong Kong, Hong Kong; 6 The Research Center of Psychology and Brain Science, Guangzhou University, Guangzhou, China; 7 Department of Imaging, Chinese Traditional Medicine Hospital of Guangdong Province, Guangzhou, China; 8 The State Key Laboratory of Brain and Cognitive Science, The University of Hong Kong, Hong Kong, Hong Kong; 9 Institute of CNS Regeneration, Jinan University, Guangzhou, China; 10 Department of Ophthalmology, The University of Hong Kong, Hong Kong, Hong Kong

## Abstract

Heroin use is closely associated with emotional dysregulation, which may explain its high comorbidity with disorders such as anxiety and depression. However, the understanding of the neurobiological etiology of the association between heroin use and emotional dysregulation is limited. Previous studies have suggested an impact of heroin on diffusivity in white matter involving the emotional regulatory system, but the specificity of this finding remains to be determined. Therefore, this study investigated the association between heroin use and diffusivity of white matter tracts in heroin users and examined whether the tracts were associated with their elevated anxiety and depression levels. A sample of 26 right-handed male abstinent heroin users (25 to 42 years of age) and 32 matched healthy controls (19 to 55 years of age) was recruited for this study. Diffusion tensor imaging data were collected, and their levels of anxiety and depression were assessed using the Hospital Anxiety and Depression Scale. Our findings indicated that heroin users exhibited higher levels of anxiety and depression, but the heroin use-associated left uncinate fasciculus was only related to their anxiety level, suggesting that association between heroin and anxiety has an incremental organic basis but that for depression could be a threshold issue. This finding improves our understanding of heroin addiction and its comorbid affective disorder and facilitates future therapeutic development.

## Introduction

Heroin belongs to the family of opiates and is highly addictive because it can effectively cross the blood–brain barrier to reach brain sites faster than other substances such as morphine.^1^ This characteristic enables heroin to exert stronger euphoric and addictive effects, and has been suggested to associate with emotional dysregulation.^2^ Under normal circumstances, emotional processing is modulated by top-down executive control during emotion-evoking situations.^3^ This regulatory strategy involves the downregulation of the reactivity of the limbic system (for example, the amygdala) to negative stimuli by regions including the dorsal anterior cingulate and medial prefrontal cortices.^4^ However, when emotional regulation is compromised, anxiety and depression may become elevated, ultimately causing certain considerably maladaptive psychological outcomes, such as anxiety disorders^5, 6^ and depression.^7, 8^

Heroin use is strongly associated with emotional dysregulation, which may explain its high comorbidity with disorders such as anxiety and depression. In previous studies, anxiety sensitivity has been reported to be associated with substance use, and the prevalence of anxiety disorders among heroin users is more than half.^9, 10^ The depression symptoms experienced by heroin addicts were comparable to those of non-addicted depressed patients.^11^ The prevalence of depression among heroin addicts ranges from 16 to 44% depending on the population and the setting.^12^ Although these comorbidities are clearly associated with morbidity, mortality, increased risk of overdose, suicide incidence and relapse rate,^13, 14, 15^ our understanding of the neurobiological mechanisms underlying heroin use and its association with emotional dysregulation remains limited.

Several neuroimaging studies have aimed to explore heroin users' brain changes and their corresponding impairments in cognition and behavior, the results of which revealed that heroin addiction resembles a disorder of neural connectivity.^16^ Importantly, neuroimaging findings using diffusion tensor imaging (DTI) have suggested a substantial overlap between changes in white matter (WM) structural connectivity among heroin users and anxiety disorder or depression. DTI was used to investigate WM structural connectivity by quantifying the degree and directionality of diffusion of water molecules along their fiber tracts and local neural tissue via four diffusion indices. In general, fractional anisotropy (FA) has been suggested to characterize WM integrity, mean diffusivity (MD) has been suggested to characterize changes in diffusion tensors, and axial diffusivity (AD) and radial diffusivity (RD) have been suggested to be specific for axonal and myelination changes, respectively.^17^

Using DTI, studies have revealed aberrant WM structural connectivity in chronic heroin users and have associated these WM microstructural damages with their impairments in risk taking,^18^ emotional regulation and memory.^19^ These widespread disruptions in WM connectivity were consistently observed in the WM connecting the frontal, cingulate, corpus callosum and temporal regions, with more widespread damage among those with more prolonged heroin usage.^18, 20, 21, 22^ In addition, individuals with generalized anxiety disorder or major depressive disorder often exhibit differences in WM connections involving frontal and limbic regions; for instance, the uncinate fasciculus that connects frontal regions to temporal regions, including the amygdala, was reported to be different.^23, 24, 25^ Patients with social anxiety disorder were reported to exhibit anomalies in WM and deficits in the left insula and the left inferior frontal gyrus were associated with the severity of social anxiety.^26^ At the subclinical level, individual differences in trait anxiety were also associated with WM connectivity in the left temporal cortex and the uncinate fasciculus.^27, 28, 29^ Specific impairments were observed in the corpus callosum and the uncinate fasciculus in adolescence-onset patients.^30^ WM matter deficits in connections involving cingulate region and the parahippocampal area were identified in young adult^31^ and late-life depression patients.^32, 33^ The severity of depression was suggested to be associated with the WM deficits in connectivity involving the frontal, limbic and cingulate cortices and the corpus callosum, including WM deficits in the anterior cingulate cortex in subclinical depression.^34^ These studies have indicated close associations between WM connectivity, specifically in the uncinate fasciculus, and the levels of anxiety and depression in both subclinical and clinical populations. Particularly, the overlap of the changes in structural connectivity between heroin use and anxiety disorder and depression suggests that the effects of heroin on structural connectivity might disrupt emotional regulation. Therefore, the next logical step would be to investigate whether the effect of heroin on the neurobiological emotional regulatory system is general or specific. We hypothesized that (1) the microstructural WM in frontal, cingulate and temporal regions in heroin users are associated with their duration of heroin use and that (2) the connectivity between these regions, as reflected by diffusivity indices, are related to the severity of both anxiety and depression in heroin users.

## Materials and methods

### Participants

Twenty-six male abstinent heroin users with mean age of 34.23 years (s.d.=3.85) and thirty-two age-matched healthy controls with mean age of 31.84 years (s.d.=11.08) participated in this study, which was approved by the Institutional Review Board of The University of Hong Kong and the Hospital Authority (The West Cluster). The heroin users were recruited from two drug rehabilitation centers, and those who had any history of replacement therapy were excluded from the present study to avoid medication effects.^19^ As polydrug use was very common among drug abusers, our heroin subject group was defined as those using heroin at >50% of their overall drug use and diagnosed with heroin abuse or dependence based on a structured clinical interview. Age-matched healthy controls without any history of substance abuse were recruited using advertisements in the local communities. No participants exhibited any neurological or psychiatric disorder. Raven's Progressive Matrices,^35^ a test of nonverbal intelligence, was administered to the participants to ensure that the intellectual capacity of the heroin users and the healthy controls was matched. Their body mass index was also measured and matched accordingly. This study was performed in accordance with the Declaration of Helsinki. Written informed consent was obtained from all the participants.

### Behavioral measures

The Hospital Anxiety and Depression Scale (HADS) was used in this study.^36^ This scale consists of 14 items on a four-point Likert scale measuring the level of distress in the subjects. Two separate scores of their anxiety and depression levels were obtained and analyzed in this study.

### DTI data acquisition and preprocessing

DTI data sets were obtained using a 3T GE Signa Propeller HD MR Scanner (GE Healthcare, Milwaukee, WI, USA) equipped with a standard multichannel head coil. They were acquired in 25 diffusion gradient directions with a nondiffusion weighted (*b*=0) reference using the following imaging parameters: TR=10 000 ms, TE=88.4 ms, flip angle=90°, phase encoding direction=COL and voxel size=0.94 × 0.94 × 4.00mm. T1-weighted structural images were also obtained, and the corresponding parameters were: TR=9.464 ms, TE=3.892 ms, flip angle=20°, phase encoding direction = ROW and voxel size = 0.90 × 0.47 × 0.47 mm. The anterior commissure, the posterior commissure and the mid-sagittal plane were manually defined in the T1-weighted images and were used to convert the T1-weighted images to the conventional anterior commissure–posterior commissure-aligned space via rigid-body transformation.

All DTI data were preprocessed using the VISTA Lab (Vision Imaging Science and Technology Lab, Stanford University) diffusion MRI software. The DTI images were first aligned to the *b*=0 image and were re-sampled into 2 × 2 × 2 mm voxels with the eddy-current correction, motion correction and anatomical alignment transformation (that is, to the anterior commissure–posterior commissure-aligned T1-weighted image of the same subject) using a seventh-order b-spline algorithm. Then, the diffusion tensors were fitted to the re-sampled DTI data using a robust least-squares algorithm and outliers from the diffusion tensor estimation were removed. From the diffusion tensor model, four diffusivity measure maps for each subject were computed. FA and MD are summative measures that describe a normalized s.d. of the three diffusion directions and average total diffusion, respectively; whereas AD and RD describe diffusion parallel and perpendicular to the principal diffusion direction, respectively. The units for MD, AD and RD would be mm^2^s^−^^1^. The four diffusivity maps (FA, MD, AD and RD) of each subject were further masked, of which any undefined values in the diffusivity maps were excluded using FMRIB Software Library v5.0.^37^

### Statistical analysis

Welch's *t*-tests were used to compare the characteristics and the HADS scores between the groups. For the DTI data sets, voxel-wise statistical analysis of the resulting FA, MD, AD and RD maps were performed using Tract-Based Spatial Statistics (TBSS)^38^ in FMRIB Software Library v5.0. TBSS, a method of voxel-wise statistics that is performed on an alignment-invariant tract representation, was used to investigate the association between heroin use and the anxiety and depression levels in our sample. This method is more sensitive and objective for analyzing diffusion images than the conventional structural voxel-based morphometry analysis, as the latter is susceptible to the generation of false positives.^39^ All the FA images were aligned to the target FA image and were transformed to the standard Montreal Neurological Institute (MNI) space during nonlinear registration. Then, a mean FA image was produced and thinned to generate a mean FA skeleton using FA >0.2 as the threshold. Next, the individuals' FA, MD, AD and RD maps were projected onto the mean FA skeleton, and all diffusivity maps were subsequently used for voxel-wise statistical analyses for comparisons between the abstinent heroin users and the healthy controls (see Supplementary Information). The associations between all diffusivity maps and the duration of heroin use or the duration of abstinence in the heroin users were also investigated using voxel-wise statistical analysis. All the analyses were based on 5000 permutations and were carried out within the mean FA skeleton mask with adjustment of covariance. A 0.05 significance level was adopted after correction for family-wise error (FWE) using threshold-free cluster enhancement.

On the basis of the findings concerning the duration of heroin use by TBSS, tracts of interest (TOIs) were identified according to the probabilistic tractography atlas (the Johns Hopkins University WM tractography atlas). The age, the Raven's Progressive Matrices score, the duration of abstinence and the average diffusivity indices (that is, FA, MD, AD or RD) for each TOI were demeaned and then included as independent variables in multiple linear regression models and the HADS anxiety and depression scores as dependent variables for further analyses of the heroin users. It was performed to investigate how the heroin users' HADS anxiety or depression levels changed when the diffusivity of a specific TOI varied.

## Results

### Group characteristics

The age, body mass index, and Raven's Progressive Matrices score of the heroin users and the healthy controls were matched (*P*>0.05; Table 1). The users on average have used heroin for 63.38 months (s.d.=67.30) and were abstained for 12.46 months (s.d.=9.94).

Heroin users were also found to experience elevated levels of anxiety (users: M=14.58, s.d.=3.71; controls: M=5.91, s.d.=3.32; *t*=9.27, *P*<0.001) and depression (users: M=14.00, s.d.=2.15; control: M=4.69, s.d.=3.09, *t*=13.47, *P*<0.001).

### Associations between the heroin consumption and WM tract characteristics in heroin users

**Figure 1:**
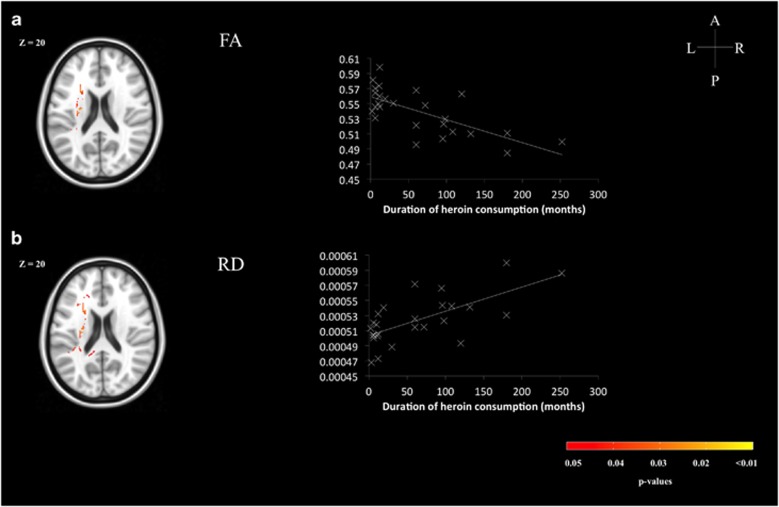
WM tract clusters in heroin users that were significantly associated with their duration of heroin consumption in months after controlling for age, Raven's Progressive Matrices score and duration of abstinence are overlaid in red-yellow on the corresponding template in standard MNI space. (**a**) FA; (**b**) RD. The mean FA and RD of the significantly different clusters are plotted against the duration of heroin consumption for demonstration purposes. A, anterior; FA, fractional anisotropy; L, left; MNI, Montreal Neurological Institute; P, posterior; R, right; RD, radial diffusivity; WM, white matter.

General linear models were set up to determine the associations between heroin use and WM tract characteristics using TBSS. Negative associations were observed in FA of some WM clusters, peaking at the left superior longitudinal fasciculus (*k*=2172, MNI coordinates: *x*=−26, *y*=−1, *z*=31, *T*=4.68, *P*<0.05 FWE-corrected; Figure 1a), whereas positive associations were observed in MD clusters peaking at left uncinate fasciculus (*k*=168, MNI coordinates: *x*=−30, *y*=46, *z*=−2, *T*=5.13, *P*<0.05 FWE-corrected) and in RD of clusters peaking at left superior longitudinal fasciculus (*k*=5017, MNI coordinates: *x*=−35, *y*=−39, *z*=11, *T*=6.57, *P*<0.05 FWE-corrected). RD of clusters within uncinate fasciculus was also positively associated with the heroin use (*k*=446, MNI coordinates: *x*=−30, *y*=46, *z*=−2, *T*=5.14, *P*<0.05 FWE-corrected; Figure 1b). A summary of the peak voxels in the significantly different WM clusters is presented in Table 2.

### Associations between WM TOIs and the Hospital Anxiety and Depression Scale scores in heroin users

Eight WM TOIs were selected (that is, all in the left hemisphere, including the forceps major, the anterior thalamic radiation, the cerebrospinal tract, the inferior fronto-occipital fasciculus, the inferior longitudinal fasciculus, the superior longitudinal fasciculus and its temporal portion and the uncinate fasciculus) according to the peak voxels in the WM clusters, which were identified to be associated with the duration of heroin use based on TBSS. The age, the Raven's Progressive Matrices score, the duration of abstinence and the average diffusivity measures for the given TOI (that is, FA, MD, AD or RD) were included as independent variables in multiple linear regression models of the HADS anxiety and depression scores for further analyses of the heroin users. It was found that the anxiety level significantly changes when MD of the left uncinate fasciculus varied (*b*=77 909.239, *P*=0.022, *r*^2^=0.3049; Table 3). However, none of the diffusivity indices of the WM tracts were related to the depression levels in heroin users (*P*>0.05). No significant association was observed between the AD or the RD of the TOIs and the HADS anxiety or depression score (*P*>0.05).

## Discussion

To the best of our knowledge, this is the first study that mapped the WM microstructural correlates of abstinent heroin users to their anxiety and depression levels. Using DTI, widespread WM microstructural anomalies in areas including the frontal, cingulate and temporal regions were associated with the duration of heroin use in our sample, supporting our first hypothesis. On the basis of analysis of the association between the HADS anxiety and depression scores and the DTI data, only the diffusivity in the left uncinate fasciculus was significantly associated with the anxiety level of heroin users, but none of the WM tracts were related to the depression level, which did not support our second hypothesis.

### Heroin use is associated with microstructural changes in the WM tracts

Tracts involving frontal (for example, superior longitudinal fasciculus) and temporal (for example, uncinate fasciculus) regions were found to be associated with the duration of heroin use. In previous studies, reduced FA in bilateral frontal sub-gyral regions were identified in current heroin users;^20, 21^ furthermore, additional abnormalities were identified in the right temporal area.^22^ Reduction in FA in the right orbito-frontal, bilateral temporal and right parietal WM were detected in current heroin users who were heroin-dependent for less than 10 years, with more widespread damage in those who were heroin-dependent for a longer period.^40^ Our findings were also consistent with another study reporting that FA of the frontal and temporal lobule in abstinent heroin users was not significantly different from that of controls, but heroin-induced WM damage to the frontal regions remained detectable via other analyses.^41^ Moreover, controlling for abstinence duration, our association analysis between the duration of heroin use and the diffusivity of WM clusters throughout the brain in our heroin sample revealed that the WM tracts, which were vulnerable to prolonged heroin use, were related to the decreased WM integrity and the abnormal myelination, as reflected by reduced FA and increased RD, respectively. Therefore, our findings suggested that prolonged heroin use was associated with myelination changes across frontal and temporal structural connectivity.

### Diffusivity in the uncinate fasciculus relates to anxiety in heroin users

Our heroin user sample, relative to the healthy control sample, exhibited higher anxiety and depression levels. This result is in accordance with previous studies that reported a high comorbidity between heroin use and anxiety and depression.^10, 12^ Our findings suggested that the change in the left uncinate fasciculus related to prolonged heroin use was associated with the level of anxiety of heroin users. The uncinate fasciculus, which connects the anterior temporal lobe (including amygdala) to the medial and lateral orbitofrontal cortex (OFC), is particularly important for emotional processing and regulation.^42, 43^ Patients with social anxiety disorder were reported to display WM anomalies in the inferior frontal gyrus and the middle temporal gyrus,^26^ and individual differences in trait anxiety were also associated with deficits in WM connectivity in the uncinate fasciculus.^27, 28, 29^ Along these lines, the impairment in the uncinate fasciculus observed in our heroin sample may explain the emotional dysregulation exhibited by heroin abusers^19, 44^ and the alteration in the reward circuit characterized by drug dependence.^45, 46^

In a previous study, substance addicts with affective disorder displayed significantly reduced brain activity in their right frontal and left temporal regions.^47^ Within the temporal region, the pivotal role of the amygdala in drug addiction has been illustrated,^48^ and studies have reported that heroin acutely affects negative emotional processing and regulation by modulating amygdala responses.^44, 49^ Our present findings confirmed widespread damage to WM microstructures due to heroin use, and this damage was located in not only the amygdala but also its projections to OFC regions, which was in accordance with another study that reported that opioid-dependent patients exhibited alterations in the affective network, including connections projected from the amygdala.^50^ The OFC has a key role in responses to reinforcement by evaluating rewarding and punishing reinforcers.^51, 52^ One study has investigated heroin addicts using PET and observed that the regional cerebral blood flow in the OFC was associated with their urge to use heroin.^53^ Abnormal patterns of fMRI activation in the bilateral OFC of heroin users during decision-making were reported; furthermore, a strong association between the duration of heroin use and the activation of their left OFC was detected.^54^ The MD of the uncinate fasciculus in our heroin users associated with their anxiety level, indicating the close association between prolonged heroin use-related microstructural changes in these WM tracts and their anxiety level. Our finding supports the concept that heroin use may be associated with the users' elevated anxiety level by damaging the WM in the uncinate fasciculus, although this speculation requires verification in future studies.

In contrast, heroin users' depression level did not change while the diffusivity of WM tracts varied. In previous reports, only few animal studies have reported on the heroin-induced anxiety or depression levels.^55, 56^ Our findings suggest that the prolonged heroin use-induced changes in WM tract diffusivity were associated with elevated anxiety on an incremental organic basis but not with depression. We propose that heroin use may induce elevated anxiety by affecting the uncinate fasciculus, whereas elevated depression may be observed in heroin users when sufficient dosages of heroin were taken.

Stress hormone release with respect to acute heroin use was evaluated recently and a close association between reduced left amygdala responses, lower anxiety levels and lower concentrations of stress biomarkers, such as adrenocorticotropic hormone, serum cortisol and saliva cortisol, was identified after administration of heroin.^49^ Heroin users might ultimately adapt to the dampening of anxiety by continuously using heroin and might develop an unhealthy mechanism of managing increased anxiety, paired with structural changes in underlying brain connectivity. Therefore, the elevated anxiety was related to changes in the uncinate fasciculus that was associated with chronic heroin use, as demonstrated in our study. However, prospective studies are required to confirm these results and to further investigate the relationship between depression and heroin use.

On the basis of the findings of this study, heroin users exhibited compromised emotional regulation, as reflected by their elevated anxiety and depression levels. Particularly, elevated anxiety was related to the uncinate fasciculus that was associated with heroin use. The uncinate fasciculus connects frontal to temporal regions and is responsible for top-down emotional regulation.^43^ Therefore, the current findings pointed to the direction that prolonged heroin use may damage users' uncinate fasciculus, which is related to aberrant top-down emotional regulation. Such heroin-induced emotional dysregulation depended on the users' elevated anxiety; however, the elevated depression levels experienced by the heroin users appeared to be more of a threshold issue. Our findings also support the theoretical model explaining how heroin intake increases anxiety via cognitive and biopsychological mechanisms.^57^ We believe that our findings have increased our understanding of substance addiction and its comorbid affective disorder, facilitating future therapeutic development for heroin abuse.

## Figures and Tables

**Table 1:** Group characteristics of heroin users and controls

*Group characteristics*	*Heroin users* (n=*26)*	*Controls* (n=*32)*	*Statistical analysis*	
			t	P
Age (years)	34.23 (3.85)	31.84 (11.08)	1.14	NS
Body mass index	22.41 (2.04)	23.28 (3.03)	1.30	NS
Raven's Progressive Matrix	43.23 (8.21)	45.69 (8.89)	1.09	NS
Education (years)	8.46 (1.65)	11.16 (2.82)	4.53	<0.001
Alcohol (drinks per day)	1.87 (1.87)	0.09 (0.53)	4.67	<0.001
Cigarettes (sticks per day)	18.27 (5.31)	3.28 (5.06)	10.92	<0.001
HADS-anxiety	14.58 (3.71)	5.91 (3.32)	9.27	<0.001
HADS-depression	14.00 (2.15)	4.69 (3.09)	13.47	<0.001
Duration of chronic heroin consumption (months)	63.38 (67.30)	—		
Duration of heroin dependent (months)	13.19 (18.80)	—		
Duration of abstinence (months)	12.46 (9.94)	—		

Abbreviations: HADS, Hospital Anxiety and Depression Scale; NS, not significant.

Welch's *t*-tests were used. The results are expressed as the means (s.d.).

**Table 2:** WM clusters associating with heroin use

*Tracts/Regions*		*Laterality*	*Coordinate*	*No. of voxels*	t*-value*
*Negative association*					
FA	SLF	L	−26, −1, 31	2172	4.68
	CST	L	−4, −29, −33	425	4.63
	CST/ATR	L	−10, −28, −18	318	5.08
	ILF/IFOF/SLFt/SLF	L	−35, −39, 11	242	4.52

*Positive association*					
MD	IFOF/ATR/UF	L	−30, 46, −2	168	5.13
RD	ILF/IFOF/SLFt/SLF	L	−35, −39, 11	5017	6.57
	IFOF/ATR/UF	L	−30, 46, −2	446	5.14
	FMA	L	−11, −41, 19	184	4.18
	ATR	L	−24, −49, 29	64	2.87
	ILF	L	−22, −53, 31	57	3.25
	SLF/SLFt	L	−31, −6, 17	16	5

Abbreviations: ATR, anterior thalamic radiation; CST, corticospinal tract; FA, fractional anisotropy; FMA, forceps major; IFOF, inferior fronto-occipital fasciculus; ILF, inferior longitudinal fasciculus; L, left; MD, mean diffusivity; R, right; SLF, superior longitudinal fasciculus; SLFt, temporal division of the superior longitudinal fasciculus; UF, uncinate fasciculus; WM, white matter.

Peak voxel of each WM cluster is shown. Age and Raven's Progressive Matrices score were included as covariates using a statistical threshold of *P*<0.05 after family-wise error correction. The coordinates were in Montreal Neurological Institute (MNI) space.

**Table 3:** Multiple linear regression analyses of TOI-predicting HADS scores

*Dependent variable*	*Independent variable*			
			b_*Diffusivity indices of TOI*_	r^*2*^
*HADS-anxiety*				
	FA	FMA	−41.803	0.1114
		L ATR	19.656	0.1055
		L CST	22.284	0.1063
		L IFOF	−18.367	0.1046
		L ILF	−7.432	0.1037
		L SLF	−122.211	0.1403
		L SLFt	−90.204	0.1327
		L UF	−8.737	0.1037
	MD	FMA	−58 062.589	0.2195
		L ATR	50 499.575	0.2311
		L CST	−4269.317	0.1083
		L IFOF	1144.624	0.1035
		L ILF	−61 504.241	0.1805
		L SLF	−1770.156	0.1036
		L SLFt	1090.807	0.1035
		L UF	77 909.239*	0.3049

*HADS-depression*				
	FA	FMA	−19.276	0.1904
		L ATR	−17.440	0.1901
		L CST	22.581	0.1939
		L IFOF	−14.795	0.1876
		L ILF	3.361	0.1855
		L SLF	11.759	0.1865
		L SLFt	17.889	0.1889
		L UF	−26.756	0.1917
	MD	FMA	9305.729	0.1943
		L ATR	13 905.994	0.2141
		L CST	4041.296	0.1981
		L IFOF	4091.053	0.1861
		L ILF	−1382.170	0.1856
		L SLF	7837.844	0.1912
		L SLFt	1212.168	0.1855
		L UF	8620.549	0.1928

Abbreviations: ATR, anterior thalamic radiation; CST, cerebrospinal tract; FA, fractional anisotropy; FMA, forceps major; HADS, Hospital Anxiety and Depression Scale; IFOF, inferior fronto-occipital fasciculus; ILF, inferior longitudinal fasciculus; L, left; MD, mean diffusivity; R, right; SLF, superior longitudinal fasciculus; SLFt, temporal division of the superior longitudinal fasciculus; TOI, tract of interest; UF, uncinate fasciculus.

The *b* (the unstandardized coefficients) and *r*^2^ values (portion of variance explained) from multiple linear regression analyses of FA and the MD of TOIs for predicting the HADS anxiety and depression scores in heroin users are presented after controlling for age, Raven's Progressive Matrices score and abstinence.

^*^P<0.05.

## Supplementary Material

Supplementary Information

Supplementary Figure1

Supplementary Figure2
