# Supplementary material for: Diffusivity of the uncinate fasciculus in heroin users relates to their levels of anxiety
Source: Transl Psychiatry. 2015 Apr 28;5(4):e554–. doi: 10.1038/tp.2015.48 (PMC4462611; doi:10.1038/tp.2015.48)
Supplement: Supplementary Information [file tp201548x1.doc]

**Supporting Information**

**SI Results**

***Associations between abstinence periods and WM tract characteristics in heroin users***

The FA of clusters in the forceps minor, the left cingulum near the cingulate gyrus and the body of the corpus callosum was positively associated with the duration of abstinence (Figure S2).

***Group comparisons of the WM tracts***

Voxel-wise comparisons of the diffusivity of the white matter (WM) tracts between the heroin users and control groups were conducted using Tract-Based Spatial Statistics (TBSS). Fractional anisotropy (FA) of clusters in the body of the corpus callosum in the heroin users was higher than that in the healthy controls (k=240, MNI coordinates: x=15, y=8, z=32, T=3.99, p<0.05 FWE-corrected; Figure S1a). Alternatively, lower MD, AD and RD in a widespread of tracts in heroin users was observed, peaking at forceps minor (MD: k=43737, MNI coordinates: x=-17, y=41, z=1, T=5.63; AD: k=21260, MNI coordinates: x=14, y=38, z=32, T=5.52; RD: k=18407, MNI coordinates: x=-17, y=41, z=1, T=5.56, all p<0.05 FWE-corrected; Figure S1b, c, d). A summary of the peak voxels in the significantly different WM clusters is presented in Table S1.

**SI Discussion**

***Recovery of WM tracts after abstinence***

To date, few studies have been conducted on brain structural and WM microstructural deficits in heroin users after prolonged abstinence. Our findings show that the WM integrity of clusters in the body of corpus callosum in heroin users was higher than that in healthy controls. Our association analysis between the duration of abstinence and the diffusivity of WM clusters throughout the brain in our heroin sample revealed that the corpus callosum, the forceps minor and the left cingulum near the cingulate gyrus were associated with the duration of abstinence. These findings were largely consistent with another study,^1^ who reported significant recovery of the corpus callosum in heroin-addicts. We also observed recovery of the forceps minor extending from the anterior portion of the corpus callosum,^2^ which projects fibers from the prefrontal cortex, the rostral cingulate cortex, and the premotor and supplementary motor areas across hemispheres.^3^

We propose two possible reasons for the observed significant associations between the WM clusters and the abstinence duration. First, a prolonged period of abstinence from heroin use improves WM integrity in the cingulate and connected frontal regions. Former heroin addicts after a period of abstinence were reported to recover inhibitory control, exhibiting no behavioral difference compared to healthy controls.^4^ Both the forceps minor and the corpus callosum are strongly related to inhibitory control.^5^ Therefore, it is very likely that abstaining from heroin use leads to the recovery of the underlying WM microstructures in these tracts and the observed behavioral improvement, although the inhibitory control of heroin users was not evaluated in the current study. While heroin addicts displaying greater WM integrity in these regions (i.e., those related to inhibitory control) may be more capable of maintaining abstinence for a longer period,^6^ we cannot draw such a conclusion based on the current results because the heroin users recruited for this study were abstaining from heroin at the time of participation. The duration of abstinence in the current study may only represent how long they had abstained from heroin use rather than how long they could maintain abstinence. Therefore, based on our findings, we speculate that a period of approximately one year of abstinence from heroin use results in reversible microstructural changes primarily in the forceps minor and the corpus callosum.

It was also worth noting that we were unable to replicate any findings of reduced FA that previous studies have reported. One possible reason for the inconsistent FA findings between our study and other studies could be related to the heroin user sample that we enrolled. We recruited abstinent heroin users because we aimed to investigate the long-term effects of heroin use on WM structural connectivity, whereas most previous studies have recruited current heroin users^7,8,9^ or users with a shorter duration of abstinence.^10^ In addition, we have excluded heroin users who had a history of replacement/methadone therapy to avoid potential medication effects.^11^

**SI References**

1. Shen X, Wang E, Wang X, Lou M. Disrupted integrity of white matter in heroin-addicted subjects at different abstinent time. *J Addict Med* 2012; **6**: 172-76.

2. Catani M, de Schotten MT. A diffusion tensor imaging tractography atlas for virtual in vivo dissections. *Cortex* 2008*;* **44**: 1105-32.

3. Schmahmann JD, Smith EE, Eichler FS, Filley CM. Cerebral white matter: neuroanatomy, clinical neurology, and neurobehavioral correlates. *Ann N Y Acad Sci* 2008; **1142**: 266-309.

4. Morie KP, Garavan H, Bell RP, De Sanctis P, Krakowski MI, Foxe JJ. Intact inhibitory control processes in abstinent drug abusers (II): a high-density electrical mapping study in former cocaine and heroin addicts. *Neuropharmacology* 2014; **82**: 151-60.

5. Olson EA, Collins PF, Hooper CJ, Muetzel R, Lim KO, Luciana M. White matter integrity predicts delay discounting behavior in 9- to 23-year-olds: a diffusion tensor imaging study. *J Cogn Neurosci* 2009; **21**: 1406-21.

6. Connolly CG, Foxe JJ, Nierenberg J, Shpaner M, Garavan H. The neurobiology of cognitive control in successful cocaine abstinence. *Drug Alcohol Depend* 2012; **121**: 45-53.

7. Qiu Y, Jiang G, Su H, Lv X, Zhange X, Tian J. Progressive white matter microstructure damage in male chronic heroin dependent individuals: A DTI and TBSS study. *PLoS ONE* 2013, **8**, e63212.

8. Liu H, Li L, Hao Y, Cao D, Xu L, Rohrbaugh R. Disrupted white matter integrity in heroin dependence: A controlled study utilizing diffusion tensor imaging. *Am J Drug Alcohol Abuse* 2008; **34**: 562-75.

9. Li W, Li Q, Zhu J, Qin Y, Zheng Y, Chang H, *et al*. White matter impairment in chronic heroin dependence: A quantitative DTI study. *Brain Res* 2013; **1531**: 58-64.

10. Wang Y, Li W, Li Q, Yang W, Zhu J, Wang W. White matter impairment in heroin addicts undergoing methadone maintenance treatment and prolonged abstinence: A preliminary DTI study. *Neurosci Lett* 2011; **494**: 49-53.

11. Lin W-C, Chou K-H, Chen C-C, Huang C-C, Chen H-L, Lu C-H, et al. White matter abnormalities correlating with memory and depression in heroin users under methadone maintenance treatment. *PLoS ONE* 2012; **7**: e33809.

**SI Figure Legends**

**Figure S1.** WM tract clusters significantly different between heroin users and controls after controlling for age and Raven’s Progressive Matrices score are shown and are overlaid in Red-Yellow on the corresponding template in standard MNI space. (a) FA (*Heroin users > Controls*); (b) MD (*Controls > Heroin users*); (c) AD (*Controls > Heroin users*) and (d) RD (*Controls > Heroin users*). A=anterior, L=left, P=posterior, R=right.

**Figure S2.** FA of WM tract clusters in heroin users that were significantly associated with their duration of abstinence in months after controlling for age, Raven’s Progressive Matrices score and duration of heroin consumption are overlaid in Red-Yellow on the corresponding template in standard MNI space. The mean FA of the significantly different clusters is plotted against the duration of abstinence for demonstration purposes. A=anterior, L=left, P=posterior, R=right.

**Table S1.** **WM clusters with differences between heroin users and controls**

|  |  | |  |  |  |  |
| --- | --- | --- | --- | --- | --- | --- |
|  | **Tracts/Regions** | | **Laterality** | **Coordinate** | **No. of voxels** | **t-value** |
|  |  | |  |  |  |  |
| ***Heroin users > Controls*** |  |  | |  |  |  |
|  |  |  | |  |  |  |
| **FA** | BCC | R | | 15, 8, 32 | 240 | 3.99 |
|  |  |  | |  |  |  |
| ***Heroin users < Controls*** |  |  | |  |  |  |
|  |  |  | |  |  |  |
| **MD** | FMI/CG | L | | -17, 41, 1 | 43737 | 5.63 |
|  |  |  | |  |  |  |
| **AD** | CG/FMI | R | | 14, 38, 32 | 21260 | 5.52 |
|  |  |  | |  |  |  |
|  | SLFt/SLF | R | | 27, 7, 18 | 13278 | 5.01 |
|  |  |  | |  |  |  |
|  | SLF | R | | 35, -11, 51 | 24 | 2.93 |
|  |  |  | |  |  |  |
|  | SMGa/PoCG/  PO | R | | 55, -26, 40 | 17 | 3.83 |
|  |  |  | |  |  |  |
|  | SLF | R | | 32, -12, 42 | 10 | 3.05 |
|  |  |  | |  |  |  |
| **RD** | FMI/CG | L | | -17, 41, 1 | 18407 | 5.56 |
|  |  |  | |  |  |  |

Peak voxel of each significantly different WM cluster is shown. Age and Raven’s Progressive Matrices score were included as covariates using a statistical threshold of p<0.05 after FWE correction. The coordinates were in MNI space.

BCC = body of the corpus callosum, CG = cingulum near the cingulate gyrus, FMI = forceps minor, SMGa = anterior division of the supramarginal gyrus, SLF = superior longitudinal fasciculus, SLFt = temporal division of the superior longitudinal fasciculus, PO = parietal operculum, PoCG = postcentral gyrus, L = left, R = right.
