# Supplementary figures and images for: Diffusivity of the uncinate fasciculus in heroin users relates to their levels of anxiety
Source: Transl Psychiatry. 2015 Apr 28;5(4):e554–. doi: 10.1038/tp.2015.48 (PMC4462611; doi:10.1038/tp.2015.48)

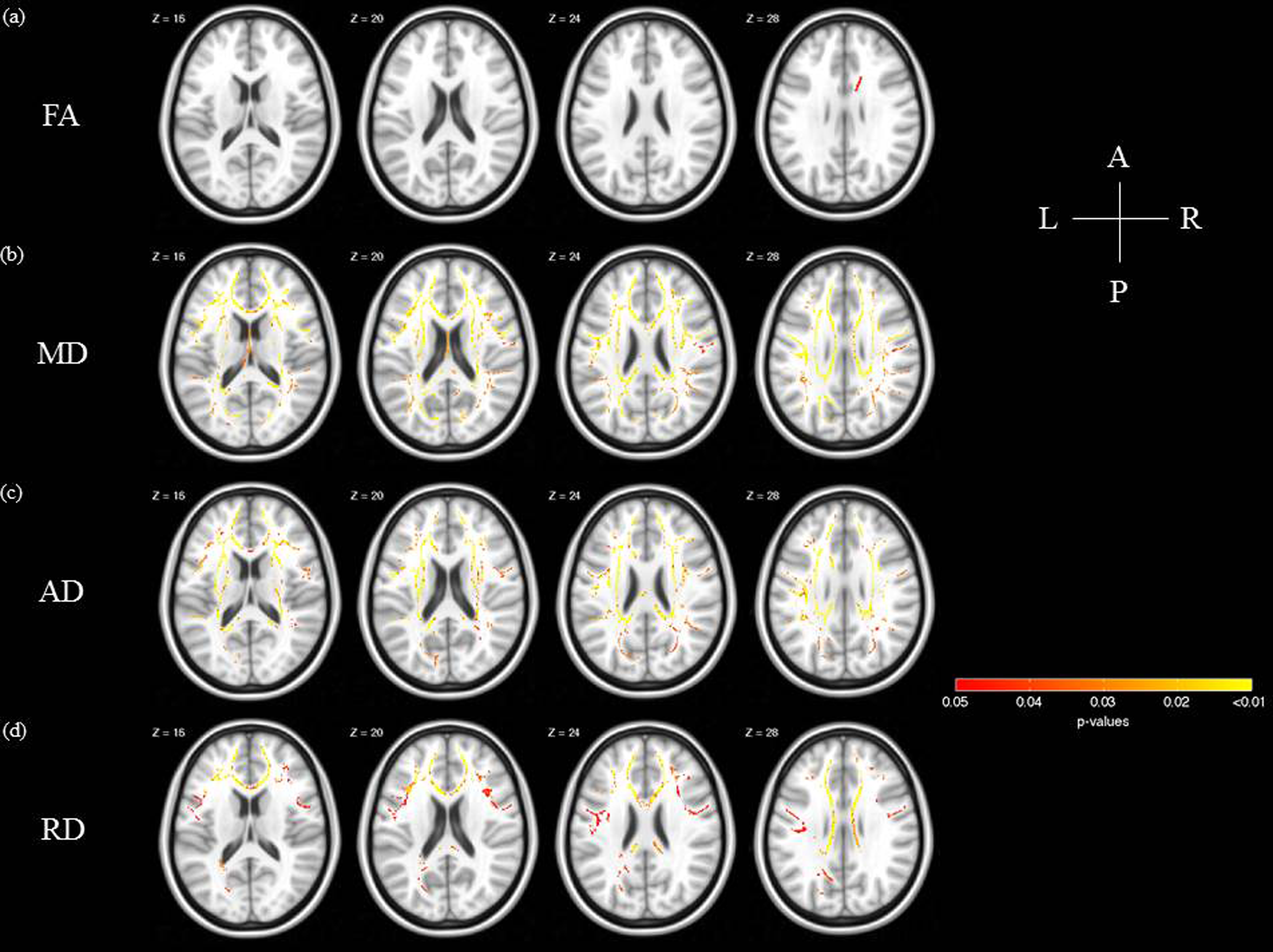

Supplement: Supplementary Figure1 [file tp201548x2.tif]

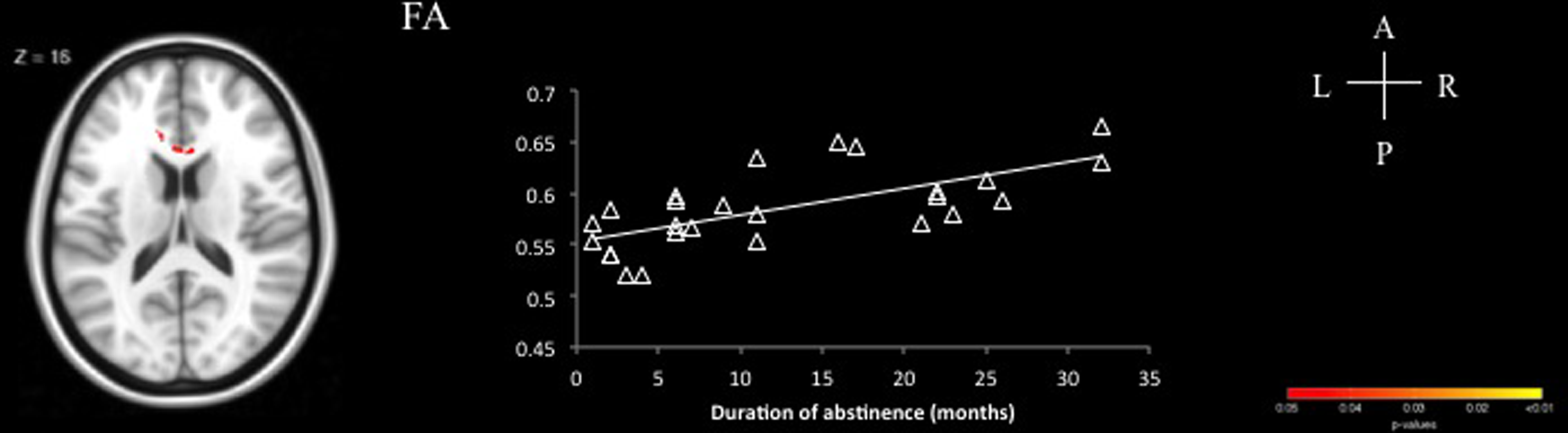

Supplement: Supplementary Figure2 [file tp201548x3.tif]
